# Maturity Has a Greater Association than Relative Age with Physical Performance in English Male Academy Soccer Players

**DOI:** 10.3390/sports9120171

**Published:** 2021-12-20

**Authors:** John M. Radnor, Jacob Staines, James Bevan, Sean P. Cumming, Adam L. Kelly, Rhodri S. Lloyd, Jon L. Oliver

**Affiliations:** 1 Youth Physical Development Centre, School of Sport and Health Sciences, Cardiff Metropolitan University, Cardiff CF23 6XD, UK; rlloyd@cardiffmet.ac.uk (R.S.L.); joliver@cardiffmet.ac.uk (J.L.O.); 2 Exeter City Football Club, Exeter EX4 6PX, UK; jacob.staines@ecfc.co.uk (J.S.); James.bevan@ecfc.co.uk (J.B.); 3 Department for Health, University of Bath, Bath BA2 7AY, UK; sc325@bath.ac.uk; 4 Faculty of Health, Education and Life Sciences, Birmingham City University, Birmingham B15 3TN, UK; Adam.Kelly@bcu.ac.uk; 5 Sport Performance Research Institute New Zealand, AUT University, Auckland 0632, New Zealand; 6 Centre for Sport Science and Human Performance, Waikato Institute of Technology, Hamilton 3200, New Zealand

**Keywords:** sprint, countermovement jump, youth, maturation, predicted adult height, football

## Abstract

This study aimed to: (1) examine differences in physical performance across birth-quartiles and maturity-status, and (2) determine the relationships among relative age, maturation and physical performance in young male soccer players. The sample included 199 males aged between 8.1 and 18.9 years, from two professional soccer academies in the English Football League. Data were collected for height, weight, self-reported biological parent heights, 30 m sprint time and countermovement jump (CMJ) height. Relative age was conveyed as a decimal, while maturity status was determined as the percentage of predicted adult height (PAH). There were no significant differences in any measure between birth quartiles, however early maturers outperformed on-time and later maturers in most performance measures. Pearson-product-moment correlations revealed that maturation was inversely associated with 30 m sprint time in U12 to U16 (*r* = −0.370–0.738; *p* < 0.05), but only positively associated with CMJ performance in U12 (*r* = 0.497; *p* < 0.05). In contrast, relative age was unrelated to sprint performance and only significantly associated with superior CMJ performance in U16. This study indicates that maturity has a greater association with sprint performance than relative age in English male academy soccer players. Practitioners should monitor and assess biological maturation in young soccer players to attempt to control for the influence on physical performance, and avoid biasing selection on absolute performance rather than identifying the most talented player.

## 1. Introduction

Soccer academies are a vital pathway in the long-term development of youth players, with the primary objective of identifying and developing talented individuals to compete at senior levels [1,2]. Two factors that have been shown to impact both player performance and selection in youth soccer are relative age and biological maturation [3,4,5]. Contrary to lay opinion, relative age and biological maturation are distinct constructs that exist and operate independent of one another [6].

*Relative age* is determined by date of birth and the selection cut-off date and refers to a player’s chronological age within their specific age group. Due to the application of arbitrary and chronologically aged (bi)annual groupings for soccer academies (e.g., U9, U10, U11, etc.), players within the same age group can be by almost twelve months apart in chronological age. This results in the phenomenon known as the relative age effect (RAE), where players born earlier in their selection year (e.g., birth quartile [BQ] one and BQ2) have a greater likelihood of being selected into talent pathways (~38–40% and ~24–30%, respectively) when compared to those born later in the year (BQ3: ~15–21% and BQ4: ~13–16%, respectively) [4,7]. 

*Biological maturation* is the process of progressing toward a mature state and varies in magnitude (extent of change), timing (onset of change) and tempo (rate of change) between different systems in the body [8] and between individuals [9]. Variance in biological maturation is a result of genetic and environmental factors and players of the same chronological age can vary by as much as five to six years in skeletal age [10], an established index of maturation in youth. As such, it is entirely possible for a player to be both the youngest and most mature player within their age group, as well as vice versa. Youth can be classified as biologically “ahead of” (early maturer), “on-time with” (average maturer) or “behind” (late maturer) their chronological age [11].

Whereas chronological age is predictable and easily assessed, biological age is significantly more difficult to assess. The gold standard method of assessing maturation is using skeletal age, but due to the expense and requirement for specialised radiographers using this method [9], other methods are often utilised. Somatic age refers to the use of growth in stature or specific dimensions of the body for the estimation of maturity [9]. The most simple level of assessment involves longitudinal anthropometric assessments [9], and the repeated collection of height over a period of time would enable the analysis of growth curves that allow information related to the initiation of the growth spurt and peak-height-velocity (PHV) to be obtained. Considering the limitations associated with collecting longitudinal data to identify PHV, predictive equations can be used to predict the age at PHV from single measurements of anthropometric variables [12,13]. Mirwald and colleagues [12] proposed a predictive equation based on the theory of differential growth rates between the lower limbs and torso. Despite this method being a popular tool for measuring maturity, it does have potential limitations. In particular, the method has received criticism from researchers who suggest a bias is prevalent towards chronological age at the time of estimation, or low sensitivity to identify early and late maturing individuals [14]. The percentage of predicted adult height (%PAH) can be calculated at a given time point during childhood and adolescence and this can be used to determine the maturational status of a young athlete [15]. This approach may be useful to differentiate between those who are early-maturing and those who are naturally predisposed to being tall, especially as it is possible that two individuals in this situation could present with the same absolute stature at a given chronological age [9]. Khamis and Roche have proposed a prediction equation to calculate final adult height, using mid-parental height but also included the child’s current stature and weight in addition to specific coefficients for each of these variables at 0.5-year intervals serve to improve the accuracy of the prediction model [16]. Recent longitudinal analysis to observe timing of PHV illustrated that %PAH was accurate 96% of the time, with maturity offset correct only 61% of the time [17]. The error of prediction in the %PAH equation has been estimated to be ~2 cm [16] and %PAH has been shown to correlate with skeletal age [18]. This has resulted in %PAH being used as a popular method of estimating maturity in youth [19] and has become increasingly popular within soccer [20] where it is used throughout the Premier League’s management application.

Differences in the maturity status and timing of individuals has been shown to have implications on the physical, psychological and athletic development of adolescent males [3,21,22]. Boys who mature in advance of their peers experience the adolescent growth spurt at an earlier age and, thus, are invariably taller and heavier from late childhood and possess greater absolute and relative lean mass [3,5,22]. As a consequence of their advanced maturity, early maturing players also tend to tend to outperform their less mature counterparts on tests of speed, power, strength, momentum, and agility [5,21]. In addition to these physical advantages, early maturing boys also tend to perceive themselves as more athletic and competent in sport [22]. Given the inherent benefits associated with advanced maturation, it is therefore not surprising that early maturing males are more likely to be represented and selected for sports where greater size, strength and power are desirable attributes, such as in soccer [3]. The selection bias towards advanced maturity in males emerges from late childhood/early adolescence and increase in size and magnitude with age and level of competition [11].

While players born earlier in the selection year are heavily represented in youth soccer [4,23], there is limited evidence to suggest that relatively older players possess advantages in functional capacities. Relatively older players are often assumed to be biologically more mature and, thus, physically superior in comparison to their relatively younger peers [24]. Despite these assumptions, relative age does not necessarily imply more advanced maturity [23], with relative age shown to be weakly correlated with maturity status in young athletes [25,26]. There is some research to suggest that players born earlier in the selection year have greater anthropometric characteristics, in addition to greater physiological attributes, which are associated with successful performance in elite youth football, however the differences between players in BQ1 and BQ4 were often unclear and predominantly trivial or small [26].

Further evidence of the independent nature of relative age and maturation can be seen in their associations with both physical and psychological variables. In a recent study investigating predictors of physical fitness in male academy soccer players, maturation was found to have a significant association with a range of physical performance measures, whereas relative age was only weakly correlated with 20 m speed and CMJ performance [27]. However, U12 to U16 were pooled together for the analysis and the effects of maturity and relative age on physical performance were not established for individual age groups. Similarly, an investigation of the ‘underdog hypothesis’ revealed that delayed maturity, but not younger relative age, was associated with greater use of adaptive self-regulated learning strategies in academy football [23]. In light of this evidence, the primary aim of the current study was to investigate the relationships between relative age, maturity and physical performance in soccer players from U9 and U18 age groups. In accordance with previous research, it was hypothesised that advanced maturation, rather than greater relative age, would be associated with superior performance on tests of sprint and jumping ability in English male academy soccer players.

## 2. Materials and Methods

### 2.1. Participants

One hundred and ninety-nine elite male junior soccer players from two professional soccer academies in the United Kingdom, between the ages of U9 and U18, volunteered to participate in the cross-sectional study. In line with the Elite Player Performance Plan (EPPP), participants trained two to four days per week, depending on age group, and typically had one competitive match per week. All players participated in a structured strength and conditioning programme, delivered by qualified coaches within the academy. Data collection occurred within the academies during the 2018–2019 and 2019–2020 seasons. None of the players reported injuries at the time of testing, nor had a major injury six months prior to testing. Parental consent and participant assent were collected for all elements of the study, in addition to a standardised health questionnaire. Ethical approval was granted by the University Research Ethics Committee for all elements of the study.

### 2.2. Procedure

#### 2.2.1. Anthropometrics

Standing height was measured using the nearest 0.1 cm with the use of a stadiometer (SECA, 321, Vogel & Halke, Hamburg, Germany). Body mass was measured to the nearest 0.1 kg on an electronic scale (SECA, 321, Vogel & Halke, Hamburg, Germany). During both anthropometric assessments, participants were instructed to stand in normal posture with weight equally distributed between feet [27].

#### 2.2.2. Birth-Date Distribution

The selection year for youth soccer in the UK spans 1st September to 31st August and consistent with previous research [4], the year was split into four quartiles. September, October and November were classified as ‘BQ1’, December, January and February classified as ‘BQ2’, March, April and May classified as ‘BQ3’, and June, July and August as ‘BQ4’. The measure of relative age was also expressed as a decimal, using the difference between a participant’s birthdate and the selection cut-off date, divided by the number of days in a year [23].

#### 2.2.3. Biological Age

To estimate biological maturation, the Khamis-Roche method was used, which requires chronological age, current height and weight of the child, and calculation of mid-parental height of the biological parents, to estimate final adult height (Equation (1)) [16]. When predicting final adult height of males between 4.0 and 17.5 years of age, the median error associated with the use of the Khamis-Roche method is 2.2 cm [16]. The standing height of participants’ biological parents was collected by academy staff or, where collection was not possible, self-reported by the parents [27]. In instances where the heights were self-reported, these were adjusted for overestimation using sex-specific equations [28] (Equation (2)).

Predicted adult height = β0 + β1 height + β2 weight + β3 mid − parent height(1)

Equation (1). Equation for predicting final adult height [16].

β0 is a sex- and age-specific intercept and β1, β2, and β3 are sex- and age-specific coefficients, in which height, weight and mid-parent height should be multiplied [29].

Male adult height (inches) = 2.316 + (0.955 × height [inches]) Female adult height (inches) = 2.803 + (0.953 × height [inches])(2)

Equation (2). Equation to adjust for self-reported heights in adults [29].

To estimate biological maturity, %PAH attained was calculated by dividing current height by PAH and multiplying by 100 [15]. Players with a greater %PAH can be expected to be more advanced in maturation compared to those further away from their PAH [27]. To estimate each participants timing of maturity, %PAH was calibrated with age- and sex-specific reference standards obtained from the UK 1990 growth reference data [30]. The age that the participants current %PAH aligned with was identified as participants biological age [31]. Maturity status was then determined using the discrepancy score between biological age (BA) and chronological age (CA). Using the traditional method of +1.0 and −1.0 for early and late maturers, respectively, fails to differentiate between individuals who differ markedly in maturity (e.g., BA-CA of +0.99 and −0.99 are both deemed on-time) [32]. Therefore, a less conservative set of criteria was applied (currently employed in the Premier League Player Management Application), and those participants with a BA-CA score of below −0.5 were classified as “late maturers”, between −0.49 and 0.49 as “on-time”, and those above +0.5 as “early maturers” [32].

#### 2.2.4. Physical Performance Tests

Countermovement Jump (CMJ): Participants performed three trials of the CMJ on a mobile contact mat (Smart Jump; Fusion Sport, Queensland, Australia), with the best jump being used for further analysis. Participants were instructed to keep their hands on their hips, and lower themselves rapidly from an initial standing position to a self-selected squat position, followed immediately by an explosive vertical jump [33]. This protocol has been reported to be a valid and reliable assessment of neuromuscular performance in youth (Intraclass correlation coefficient [ICC] = 0.83 [34]).

30 m Sprint: Sprint times during three trials of maximal sprinting over 30 m were assessed using photo-electric timing gates (Smart Speed, Fusion Sport, Queensland, Australia) on an outdoor 3G pitch. The timing gates were placed at 0 m, 5 m, 10 m, 20 m and 30 m. Participants were instructed to begin their sprint in a split stance on a line 50 cm from the first gate, to avoid starting the timer early when in their set position. Participants were then instructed to “get ready” and “go”, and were given verbal encouragement throughout each trial to ensure they were sprinting maximally through the final timing gate. A minimum of four minutes passive rest was given between trials to ensure sufficient recovery [35]. The best 30 m time was used for further analysis.

### 2.3. Statistical Analyses

The assumption of normality was assessed via the Shapiro-Wilk test, and descriptive statistics were calculated for all variables as mean and standard deviation (SD). Separate one-way analysis of covariance (ANCOVA) tests, with age as the covariate, were used to determine the differences in all measured variables between age groups, birth quartiles and maturity classifications, with a Bonferroni post-hoc analysis applied to identify any significant between-group differences.

Frequency counts were used to determine the number of players within each birth quartile (BQ1–4) and each maturity classification (early, on-time, late). Chi-square (χ2) analysis was then used to compare maturity distributions from within each birth quartile to what would be expected based on a normal distribution (30.3% as early and late maturers, and 38.3% as average maturers). Cramer’s V was also calculated to determine the magnitude of difference in frequency counts and interpreted as a value of 0.06–0.16 as a small effect size, 0.17–0.28 as a medium effect size and >0.29 as a large effect size [36]. Furthermore, analysis of the adjusted standardized residuals was completed to identify frequencies that were greater than 1.96 or less than −1.96 z-scores (*p* < 0.05), highlighting a significant difference to the expected distribution for each age group.

Relationships between both relative age and percentage of PAH, and CMJ jump height and split times from the 30 m sprint (0–5 m, 0–10 m, 0–20 m and 0–30 m) were assessed via Pearson’s correlation coefficients and interpreted as: <0.2 (no relationship), 0.2–0.45 (weak), 0.45–0.7 (moderate) and >0.7 (strong) based on previous recommendations [37].

## 3. Results

The descriptive statistics of each age group for height, weight, PAH, percentage of PAH (%PAH) and performance parameters including 5 m, 10 m, 20 m, 30 m speed and CMJ jump height are presented in Table 1.

Older age group players were significantly taller, heavier and more mature than the younger age groups (*p* < 0.05). However, there were no differences between the U11, U10 and U9 for height, weight, or PAH (*p* > 0.05), but the U11 were significantly more mature than the U10 and U9 (*p* < 0.05). From a physical performance aspect, older players significantly outperformed younger players across most sprint distances and in the CMJ (*p* < 0.05). However, there were no significant differences in sprint performance at any distance between U9 to U13, other than U12 being significantly faster than U10 at 20 m and 30 m. Specific differences in anthropometric and performance scores between age groups are shown in Table 1 and Table 2.

The adjusted means of each birth quartile for height, weight, PAH, %PAH and performance parameters including 5 m, 10 m, 20 m, 30 m speed and CMJ jump height are presented in Table 3. There were no significant differences between any birth quartile for any of the measured variables.

The adjusted means of each maturity classification are presented in Table 4. Early maturers were significantly taller and heavier compared with both on-time and late maturers (*p* < 0.05). From a performance aspect, early and on-time maturers significantly outperformed late maturers in 5 m,10 m, 20 m and 30 m sprint times (*p* < 0.05), but there were no differences in CMJ height between groups (*p* > 0.05).

**Figure 1:**
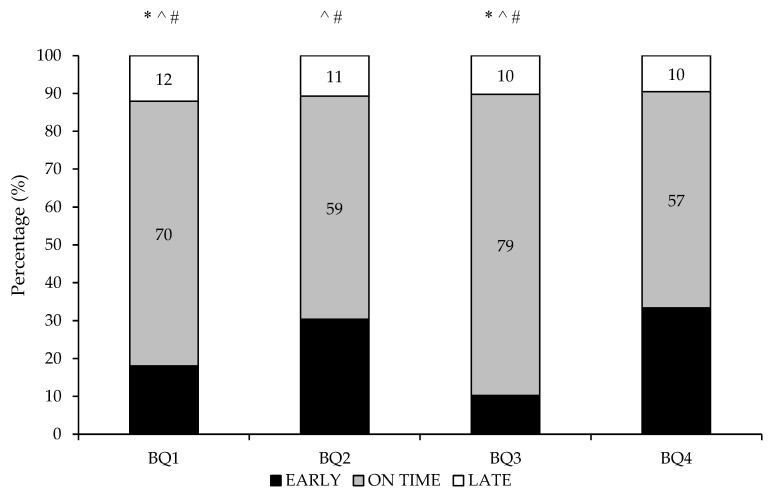
Frequency count for number of players in each maturity band from each birth quartile. * significantly fewer early maturers than expected based on normal distribution (*p* < 0.05). ^ significantly more on time maturers than expected based on normal distribution (*p* < 0.05). # significantly fewer late maturers than expected based on normal distribution (*p* < 0.05).

The maturity distributions within each birth quartile were significantly skewed with a large effect size compared to normal distribution (χ^2^ (*df* = 2) = 73.1, *p* < 0.05, *V* = 0.429) (see Figure 1). The adjusted residuals showed that there were significantly more on-time maturers and significantly less early and late maturers for the BQ1 and BQ3 than expected (*p* < 0.05).

The relationships between relative age, maturity and sprint and jump performance are displayed in Table 5. There was a significant, weak relationship between relative age and CMJ height in U16 (*r* = 0.416; *p* < 0.05), however there were no other significant associations between relative age and physical performance in any age group. U12 to U16 showed weak to strong relationships between maturity and sprint performance (*r* = 0.366 –0.711; *p* < 0.05), except for 30 m time in U13. There was also a moderate, significant relationship between maturity and CMJ height in U12 (*r* = 0.497; *p* < 0.05).

## 4. Discussion

The main finding of the current study was that maturity status and relative age were differentially associated with sprint performance in young soccer players. Specifically, advanced maturity was associated with superior sprint performance in most age groups, whereas relative age was, in the majority of cases, unrelated to sprint performance. CMJ performance was significantly associated with more advanced maturity at U12, and older relative age at U16. Collectively, these findings generally support the conclusion that advanced maturity, but not older relative age, is associated with superior sprint speed in English male academy soccer players. Thus, the arguments that relatively older players possess superior speed are not supported in this context, and the initial hypothesis can be accepted.

There were a number of significant associations observed between maturity and sprint performance in U12 to U16, however, there were no significant relationships between relative age and sprint performance in any age groups. There was also a significant association between maturity and CMJ performance in U12, whereas U16 had a significant relationship between relative age and CMJ performance. Similar findings have recently been reported, where maturity status was shown to have a much greater influence on sprint, change of direction and CMJ performance in young soccer players [27]. The findings from the current study expands on this previous research, identifying that maturity influences sprint performance between 12 and 16 years, but has limited influence prior to and after these age groups. Considering that the onset of PHV is ~85% PAH [17], the majority of players U12 and below within the current study were yet to experience their growth spurt (66/68 players < 85% PAH) and therefore may explain why maturity has no influence on sprint speed prior to this age group. Additionally, the weakest significant association between sprint performance and maturation was within the U13 age group, and this group had an average PAH of 85.7%, suggesting they were at the onset of the adolescent growth spurt. It is possible that some of the challenges associated with adapting and adjusting to the growth spurt may mitigate some of the advantages associated with advanced maturity at this stage of development.

As expected, CMJ performance significantly increased with advancing age across the entire population. However, when considered within specific age groups, which become more homogenous, relationships between maturity and CMJ were mostly non-significant. The only group where a significantly relationship did exist was for the U12, an age which is associated with the start of the growth spurt and may represent a time of more variability in maturity and performance across players [38]. Furthermore, players within the same age group would have similar resistance training ages, due to starting at the academy at the same time. These similar training ages of players may have off-set any potential benefits of advanced maturity status on CMJ performance within individual age groups.

In accordance with previous research [39,40,41], the older age groups were significantly taller, heavier and closer to their predicted adult height compared to younger age groups, while the older groups also outperformed the younger groups in sprint and jump tests. Interestingly, there were no significant differences in anthropometric characteristics between each birth quartile. This may suggest that BQ4s need to be relatively taller and heavier to be selected into soccer academies, which supports previous findings where the mean height and weight of relatively younger soccer players lay above the normal development curve, whereas the means of relatively older players lay on or under that curve [42].

Although superior values were reported across the majority of the fitness variables in players born in the first three quarters of the year compared with the last quarter, the between-group differences were not significant. As with comparable studies, these findings may be limited by the small number of BQ4 compared to the other quartiles. However, similar outcomes have been reported in previous literature [1,43], where the only difference between young players from each birth quartile was in chronological age and %PAH, with no significant difference in physical performance across birth quartiles. One explanation of these findings may be that the BQ4s who are entering into academies are better physically than the average, school-aged BQ4, and one of the reasons why they are being selected in the first instance. This could explain the lack of differences between BQ’s in performance and the lack of relationship between relative age and performance.

The current study found that early maturers were taller, heavier, faster and jumped higher than the on time and late maturers. Typically, research has reported that earlier maturing athletes have greater anthropometric characteristics (height and body mass) than later-maturing athletes [7], with previous research highlighting improvements in sprint performance with increasing maturation in young soccer players [43,44]. Cumulatively, the findings from the current study suggest that maturity status has a significant influence on sprint performance in English male academy soccer players, whereas relative age did not. As children mature, they will experience natural increases in strength and power [11], underpinned by structural and neural changes [45,46,47]. Recently, increases in muscle thickness throughout maturation were shown to be the underpinning factor in improvements in sprint speed in a cohort of school-aged boys [46]. Considering that relative maximal force is a strong predictor of sprint performance in boys [48], the increased force producing capabilities in boys as they mature may explain the influence of maturation on sprint performance.

There was a relative age bias present within the academies assessed within the current study, whereby ~70% of players were born in the first half of the year, with ~41% born in BQ1. Interestingly, although the percentage of late maturers was similar from each birth quartile (~10–12%), a greater percentage of BQ4′s were early maturers (33%) compared to the other birth quartiles (10–30%). It is often assumed that relatively older academy soccer players are further advanced in maturation and, thus, possess greater anthropometric qualities and superior performance characteristics [6]. However, the findings from the current study supports the notion that maturation and relative age are different constructs [6], and that being BQ4 does not mean that an academy soccer player will be a later maturer. However, these findings suggest that it may be important for players born in BQ4 to be early maturing to increase their likelihood of overcoming the relative age bias and being selected into an English male soccer academy. Previous research has also reported that early maturing soccer players were overrepresented in the last BQ, whereas late maturing athletes were overrepresented in the first BQ, suggesting that relatively younger soccer players may only have an opportunity of selection if they were early maturing, whereas relatively older athletes have an increased likelihood for selection independent of their biological maturity status [49,50].

Maturation influences physical performance, with early maturing boys outperforming on-time and late maturers, which has a subsequent impact on match-performance in soccer [51]. While advanced maturity offers an initial benefit in performance and selection [52], it may be detrimental in the long term, due to early maturing players neglecting their technical and tactical development in favour of using their physical prowess [52]. Research has suggested that ‘elite’ status in soccer gradually excludes early maturing boys and favour late maturing boys as age increased [53]. Those involved in the identification and development of academy players should be aware of, and accommodate for, individual differences in maturation. Bio-banding is the process of periodically grouping athletes on the basis of attributes associated with growth or maturation, rather than chronological age [38]. This approach has been used as a method to ensure holistic development of soccer players in academies and can theoretically benefit both early and late maturers, by levelling out physical requirements, ensuring that players develop technical and tactical abilities as well as using their physical qualities [20,38]. Bio-banding exists as an adjunct to, and not a replacement for, age group competition, meaning late maturing youth can also continue to experience the challenges of competing against their more mature peers in the traditional formats, which is important in the context of the underdog hypothesis [4]. Late maturing players have been found to possess superior technical skills [54] and more adaptive self-regulated learning strategies [23], and it may be important for these later maturers to compete against more mature peers in order to develop these traits that result in their success transitioning towards adulthood. One key use of bio-banding in soccer may be when comparing fitness testing data across age groups [55]. The current study has identified the influence of maturation on sprint speed, and therefore it seems prudent to identify and develop boys of the same maturational stage, as well as chronological age.

A limitation of the current study was that maturity was not assessed using the gold standard method of skeletal imaging [9]. This method requires access to specialist equipment and expertise, and is not accessible to most practitioners working in youth sport. Instead, maturity was estimated using %PAH, which is widely used in youth sport and particularly soccer, and has been shown to be a reliable method for estimating maturity [18,27]. While the current study has made a significant contribution to the literature surrounding the relationship between maturity status and performance, field-based methods were used to assess performance. Future research should attempt to collect more detailed metrics, such as force-time characteristics to better understand the influence of maturity on performance.

The RAE is well established within soccer academies, despite no clear benefit of being relatively older in terms of physical performance in those selected into an academy. Therefore, future research should aim to identify the processes and mechanisms that underpin the RAE in soccer, with a particular emphasis upon developmental attributes that afford a distinct advantage from early childhood. Moreover, the differences in physical performance outcomes between BQs who are selected into academies compared to those who are not should be explored to help better understand the role of sprint and power attributes as part of the selection process, as well as take the existing literature beyond the current academy soccer context.

## 5. Conclusions

The current study aimed to establish the relationship between maturation, relative age and physical performance. Sprint performance was associated with maturation, but not relative age, while there was no consistent relationship between relative age or maturation and CMJ performance. It is key for practitoners to understand that the RAE and maturity status are two distinct constructs, highlighted by the signifcant associaiton between sprint performance and maturation, but not relative age. Practitioners should be encouraged to monitor growth and maturation (frequent assessments of height and weight to establish predicted adult height and maturity status) to help interpret changes in physical performance of young English male academy soccer players. Furthermore, maturity status should be considered when comparing fitness scores in players to ensure practitioners are not comparing early and late maturers within the same age group, but rather are comparing boys of the same maturity status. 

## Figures and Tables

**Table 1:** Frequency count of birth quartile (BQ) and maturity classification, and descriptive statistics for anthropometric characteristics for each age group (mean ± SD).

Age Group	BQ1 (n)	BQ2 (n)	BQ3 (n)	BQ4 (n)	Early (n)	On-Time (n)	Late (n)	Height (cm)	Body Mass (kg)	PAH (cm)	%PAH
U9	4	6	2	0	2	10	0	135.6 ± 4.7	31.2 ± 2.7	178.6 ± 5.7	0.75 ± 0.01
U10	9	2	5	2	2	16	0	137.8 ± 4.8	32.7 ± 3.8	175.3 ± 5.6	0.78 ± 0.01
U11	11	5	1	0	2	11	3	143.3 ± 6.3	38.8 ± 6.1	177.0 ± 6.9	0.81 ± 0.02 ^#ª^
U12	5	7	9	1	3	18	1	148.7 ± 6.8 ^#ª^	40.8 ± 6.2 ^#ª^	178.2 ± 4.9	0.84 ± 0.02 ^^#ª^
U13	14	6	7	9	8	23	5	154.1 ± 8.4 ^^#ª^	44.6 ± 7.9 ^#ª^	180.4 ± 6.9	0.86 ± 0.03 ^^#ª^
U14	13	9	7	5	8	21	5	161.6 ± 7.5 ^¢§^#ª^	51.4 ± 9.1 ^¢§^#ª^	181.2 ± 5.5 ^#^	0.89 ± 0.03 ^¢§^#ª^
U16	15	19	6	4	14	22	8	170.7 ± 7.0 ^∞¢§^#ª^	60.0 ± 8.9 ^∞¢§^#ª^	180.3 ± 4.8 ^#^	0.95 ± 0.03 ^∞¢§^#ª^
U18	12	3	2	0	4	13	0	180.1 ± 5.3 *	75.3 ± 7.0 *	180.5 ± 5.2	1.00 ± 0.02 *

ª significantly different to U9; # significantly different to U10; ^ significantly different to U11; § significantly different to U12; ¢ significantly different to U13; ∞ significantly different to U14; * significantly different to all groups.

**Table 2:** Descriptive statistics for sprint times and CMJ height for each age group (mean ± SD).

Age Group	5 m (s)	10 m (s)	20 m (s)	30 m (s)	CMJ (cm)
U9	1.12 ± 0.04	1.96 ± 0.05	3.55 ± 0.13	5.16 ± 0.22	24.0 ± 3.5
U10	1.15 ± 0.04	2.02 ± 0.07	3.64 ± 0.14	5.27 ± 0.26	22.8 ± 2.4
U11	1.13 ± 0.06	1.99 ± 0.10	3.60 ± 0.19	5.18 ± 0.30	24.5 ± 3.4
U12	1.09 ± 0.05	1.92 ± 0.08	3.42 ± 0.18 ^#^	4.92 ± 0.27 ^#^	27.2 ± 3.7
U13	1.15 ± 0.08	1.99 ± 0.12	3.50 ± 0.20	4.96 ± 0.29 ^#^	30.6 ± 5.9 ^^#ª^
U14	1.10 ± 0.12	1.90 ± 0.14 ^¢#ª^	3.34 ± 0.21 ^¢^#ª^	4.73 ± 0.28 ^¢^#ª^	32.4 ± 5.4 ^§^#ª^
U16	1.08 ± 0.09 ^¢#^	1.85 ± 0.11 ^¢^#ª^	3.19 ± 0.16 ^∞¢§^#ª^	4.46 ± 0.22 ^∞¢§^#ª^	36.9 ± 6.2 ^∞¢§^#ª^
U18	0.99 ± 0.05 *	1.71 ± 0.06 *	2.96 ± 0.09 *	4.15 ± 0.12 *	41.9 ± 6.5 *

ª significantly different to U9; # significantly different to U10; ^ significantly different to U11; § significantly different to U12; ¢ significantly different to U13; ∞ significantly different to U14; * significantly different to all groups.

**Table 3:** Descriptive statistics for all measured variables across birth quartiles (adjusted mean ± adjusted SD).

BQ	Height (cm)	Body Mass (kg)	PAH (cm)	%PAH	5 m (s)	10 m (s)	20 m (s)	30 m (s)	CMJ (cm)
1	156.4 ± 7.1	48.5 ± 7.4	178.7 ± 5.8	87.4 ± 2.7	1.10 ± 0.08	1.92 ± 0.11	3.40 ± 0.18	4.84 ± 0.26	30.3 ± 5.4
2	158.5 ± 7.1	50.0 ± 7.3	179.7 ± 5.8	88.4 ± 2.2	1.09 ± 0.08	1.89 ± 0.10	3.35 ± 0.18	4.75 ± 0.25	31.3 ± 5.3
3	156.4 ± 7.1	47.8 ± 7.4	179.5 ± 5.8	87.1 ± 2.5	1.10 ± 0.08	1.90 ± 0.11	3.34 ± 0.18	4.73 ± 0.26	32.8 ± 5.4
4	157.8 ± 7.1	50.7 ± 7.3	181.5 ± 5.8	87.2 ± 2.3	1.14 ± 0.08	1.96 ± 0.11	3.42 ± 0.18	4.84 ± 0.26	32.1 ± 5.3

**Table 4:** Descriptive statistics for all measured variables across maturity classifications (adjusted mean ± adjusted SD).

Maturity Classification	Height (cm)	Body Mass (kg)	PAH (cm)	BA-CA (Years)	5 m (s)	10 m (s)	20 m (s)	30 m (s)	CMJ (cm)
Early	164.4 ± 6.4	56.5 ± 6.1	182.3 ± 5.9	0.89 ± 0.35	1.07 ± 0.08	1.87 ± 0.10	3.29 ± 0.18	4.69 ± 0.26	32.9 ± 5.5
On Time	156.1 ± 6.2 *	48.9 ± 5.9 *	178.7 ± 5.7 *	0.04 ± 0.29 *	1.09 ± 0.08	1.90 ± 0.10	3.37 ± 0.17	4.80 ± 0.26	30.7 ± 5.4
Late	153.3 ± 6.3 *	42.1 ± 6.0 *#	179.6 ± 5.8	−0.73 ± 0.21 *	1.16 ± 0.08 *#	2.00 ± 0.10 *#	3.49 ± 0.18 *#	4.92 ± 0.26 *#	31.7 ± 5.5

* significantly different to “early” maturers; # significantly different to “on time” maturers. BA: biological age; CA: chronological age.

**Table 5:** Pearson correlations between relative age and biological age for each age group.

Age Group	Relative Age					Maturity Status				
	5 m (s)	10 m (s)	20 m (s)	30 m (s)	CMJ (cm)	5 m (s)	10 m (s)	20 m (s)	30 m (s)	CMJ (cm)
U9	0.152	−0.179	−0.109	−0.091	−0.012	−0.264	−0.552	−0.467	−0.423	−0.291
U10	0.295	0.293	0.321	0.322	−0.077	−0.034	0.087	0.275	0.286	−0.045
U11	−0.005	−0.118	−0.105	−0.025	0.136	−0.167	−0.153	−0.163	−0.102	0.150
U12	−0.073	−0.114	−0.148	−0.146	0.216	−0.738 *	−0.655 *	−0.686 *	−0.680 *	0.497 *
U13	−0.058	−0.096	−0.052	−0.071	−0.045	−0.477 *	−0.427 *	−0.366 *	−0.291	−0.305
U14	−0.062	−0.051	−0.008	0.013	−0.222	−0.706 *	−0.711 *	−0.652 *	−0.607 *	0.026
U16	0.190	0.179	0.141	0.057	0.416 *	−0.497 *	−0.654 *	−0.609 *	−0.616 *	0.200
U18	−0.236	−0.122	−0.289	−0.272	0.265	−0.257	−0.261	−0.296	−0.299	0.348

* significant correlation (*p* < 0.05).

## Data Availability

The data is not yet publicly available.
